# Csn5 Depletion Reverses Mitochondrial Defects in GCN5-Null *Saccharomyces cerevisiae*

**DOI:** 10.3390/ijms26146916

**Published:** 2025-07-18

**Authors:** Angela Cirigliano, Emily Schifano, Alessandra Ricelli, Michele M. Bianchi, Elah Pick, Teresa Rinaldi, Arianna Montanari

**Affiliations:** 1 Department of Biology and Biotechnologies “C. Darwin”, Sapienza University of Rome, 00185 Rome, Italy; angelacirigliano82@gmail.com (A.C.); emily.schifano@uniroma1.it (E.S.); michele.bianchi@uniroma1.it (M.M.B.); teresa.rinaldi@uniroma1.it (T.R.); 2 Institute of Molecular Biology and Pathology, Consiglio Nazionale Delle Ricerche, 00185 Rome, Italy; alessandra.ricelli@cnr.it; 3 Department of Biology and Environment, Faculty of Natural Sciences, University of Haifa at Oranim, Tivon 3600600, Israel; elahpic@research.haifa.ac.il

**Keywords:** mitochondria, *Saccharomyces cerevisiae*, epigenetic regulation, lysine-acetyltransferase, ubiquitin–proteasome pathway, ergosterol

## Abstract

In this study, we investigated the mitochondrial defects resulting from the deletion of *GCN5*, a lysine-acetyltransferase, in the yeast *Saccharomyces cerevisiae*. Gcn5 serves as the catalytic subunit of the SAGA acetylation complex and functions as an epigenetic regulator, primarily acetylating N-terminal lysine residues on histones H2B and H3 to modulate gene expression. The loss of *GCN5* leads to mitochondrial abnormalities, including defects in mitochondrial morphology, a reduced mitochondrial DNA copy number, and defective mitochondrial inheritance due to the depolarization of actin filaments. These defects collectively trigger the activation of the mitophagy pathway. Interestingly, deleting *CSN5*, which encodes to Csn5/Rri1 (Csn5), the catalytic subunit of the COP9 signalosome complex, rescues the mitochondrial phenotypes observed in the *gcn5*Δ strain. Furthermore, these defects are suppressed by exogenous ergosterol supplementation, suggesting a link between the rescue effect mediated by *CSN5* deletion and the regulatory role of Csn5 in the ergosterol biosynthetic pathway.

## 1. Introduction

The lysine-acetyltransferase (KAT) Gcn5 of *Saccharomyces cerevisiae* is a key component of the Spt-Ada-Gcn5 acetyltransferase (SAGA) complex, which is a multi-subunit chromatin-modifying enzymatic complex comprising distinct functional and structural modules. Among these, the histone acetyltransferase (HAT) and deubiquitinase (DUB) modules play a central role in regulating chromatin dynamics. The activities of the HAT and DUB modules are coordinated through interactions among subunits, notably between Gcn5 and Ubp8. These two proteins associate with additional proteins to assemble the HAT module [1]. Gcn5 primarily acetylates the lysine residues present on histone H3 with established target sites including K9, K14, K18, K23, K27, and K36 [2]. In addition to histone H3, Gcn5 also modifies histones H2B and H4, as well as several non-histone substrates, such as the oncoprotein c-Myc and the metabolic coactivator PGC1α [3].

In addition to its well-established role as a nuclear epigenetic factor regulator mediating the lysine acetylation of histones H3 and H4, we have previously demonstrated that Gcn5 also contributes to mitochondrial function and is essential for efficient respiration in *S. cerevisiae* [4,5,6]. Gcn5 has been localized to the mitoplasm, the mitochondrial compartment deprived of the outer mitochondrial membrane (OMM), and is associated with the inner mitochondrial membrane (IMM) [5]. Under respiratory growth conditions, *GCN5* expression is upregulated, and accordingly, the Gcn5 protein level is elevated compared to the fermentative growth [4]. The *gcn5*Δ mutant strain exhibits compromised mitochondrial function, including abnormal mitochondrial membrane morphology, instability of mitochondrial DNA (mtDNA), and loss of mtDNA-specific regions [5,6]. It has been shown that other SAGA subunits play a role in mitochondrial function. Ubp8, a ubiquitin-specific protease belonging to the DUB module, is essential for respiration, and its depletion has been associated with mitochondrial metabolic defects [7]. Tra1 belongs to the phosphoinositide-3-kinase (PIKK) family and is an essential component of the SAGA/SLIK and NuA4 histone acetyltransferase complexes. Its genetic association with mitochondria has been demonstrated [8]. Furthermore, a *TRA1* mutant was unable to grow on a glycerol-containing medium, indicating its involvement in mitochondrial function [9].

The role of Gcn5 in epigenetic regulation is highly conserved across eukaryotic evolution [10]. Therefore, using simple model organisms such as *S. cerevisiae* offers a valuable approach for uncovering the fundamental mechanisms by which Gcn5 contributes to mitochondrial function. This could provide insight into analogous processes in higher eukaryotic organisms.

To further investigate the mitochondrial role of Gcn5, we examined the mitochondrial stability of the *gcn5*Δ mutant by monitoring the mtDNA distribution during the budding process. In yeast, mtDNA is organized into protein-associated structures known as nucleoids, which are tethered to the inner mitochondrial membrane [11,12]. During cell division, mitochondria and their associated nucleoids are actively transported from the mother cell to the bud (anterograde movement), a process that is mediated by interactions between the outer mitochondrial membrane and actin filaments [13,14].

Given that the SAGA complex is functionally connected to the ubiquitin–proteasome system, primarily through its Ubp8-containing DUB module, we explored potential genetic interactions between *GCN5* and components of the protein degradation machinery. Indeed, Ubp8 has previously been implicated in mitochondrial regulation [7], suggesting that other SAGA complex components, such as Gcn5, may similarly influence mitochondrial function through interactions with the ubiquitin–proteasome system. Notably, various proteasome subunits have been found to be involved in mitochondrial function and dynamics [11,15,16,17]. A subset of E3 ubiquitin ligases, the Cullin RING E3 ligases (CRLs), is regulated by the COP9 signalosome (CSN), a multi-subunit complex highly conserved from yeast to mammals. The CSN modulates CRL activity through deneddylation, whereby the ubiquitin-like modifier Nedd8 is removed from the Cullin subunits. This influences the degradation of numerous substrates [18]. We focused on the *CSN5* gene, which encodes the catalytic subunit of the CSN complex, for several reasons. Firstly, in contrast to higher eukaryotes, deletion of CSN subunits in *S. cerevisiae* is not lethal, which enables genetic interaction studies [19]. Secondly, it has been shown to respond to the cellular redox status during mitochondrial respiration [17]. Thirdly, it participates in a variety of cellular pathways, including the response to mating pheromones and the uptake of metal ions [20,21], as well as the regulation of genes that control amino acids and lipid metabolism [20]. Most notably, *CSN5* is involved in the ergosterol biosynthetic pathway, which is essential for the integrity and function of the mitochondrial membranes [21]. Disruption of Csn5 activity has been associated with reduced levels of ergosterol and unsaturated fatty acids (UFA), vacuolar defects, and the accumulation of endoplasmic reticulum (ER) stress [22]. Recently, Csn5 has been proposed as a potential target for fungicidal and nematocidal strategies [23,24,25].

The eukaryotic CSN and SAGA complexes play a role in development and differentiation. The *Arabidopsis thaliana* COP9 signalosome is involved in various developmental processes [26,27]. In *Drosophila melanogaster*, the loss of *GCN5* is lethal, as it prevents the progression of metamorphosis [28], whereas in mice it interferes with normal development [29]. Furthermore, both complexes are implicated in human diseases. *CSN5*/*JAB1* is overexpressed in various cancers [30,31], while *GCN5*/*KAT2* plays a role in the development of cancer, diabetes, and osteoporosis [32]. Additionally, deubiquitinase *USP22*, the human homologue of yeast *UBP8*, and *CSN5*/*JAB1* have been associated with tumour immune evasion due to their direct involvement in regulating cell surface immune receptors [33].

Here, we demonstrated that the deletion of *CSN5* suppressed the mitochondrial defects of the *gcn5*Δ mutant. Given the involvement of the Csn5 in the ergosterol biosynthesis and its relation to alterations in organelle membrane structure, the addition of ergosterol suppresses the mitochondrial phenotypes of the *gcn5*Δ mutant.

## 2. Results

### 2.1. GCN5 Gene Deletion Is Associated with mtDNA Instability

#### 2.1.1. *GCN5* Deletion Impairs mtDNA Migration into the Bud During Cell Division

**Figure 1:**
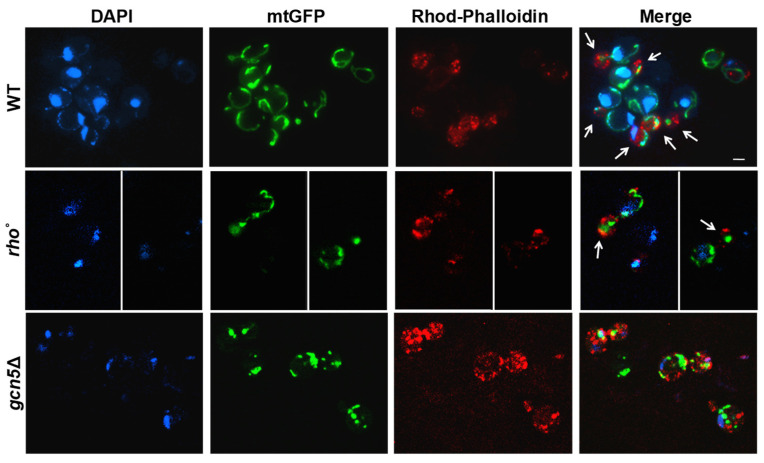
Mitochondrial morphology and movement are altered in *gcn5*Δ cells. Fluorescence microscopy of wild-type (WT), *gcn5*Δ, and *rho*° cells transformed with mtGFP (green), stained with DAPI (blue), and Rhodamine-Phalloidin (red). Fluorescent signals show mitochondria (green), DNA (blue), and the actin cytoskeleton (red). The actin polarization into the buds is indicated by arrows. In *gcn5*Δ the mitochondrial migration into the bud is reduced. Scale bars: 2 μm.

Deletion of the *GCN5* gene has previously been linked to mtDNA instability in *Saccharomyces cerevisiae* [5]. In budding yeast, mitochondrial inheritance during cell division depends on the active transport of mitochondria along actin filaments [11,12]. To assess whether *GCN5* deletion affects mitochondrial and mtDNA migration, we used fluorescence microscopy to simultaneously visualize mitochondria, actin cytoskeleton, and mtDNA. Mitochondria were labelled with mtGFP, actin filaments were stained with Rhodamine-Phalloidin, and the DNA was visualized with DAPI (Figure 1). Wild-type, *rho*°, and *gcn5*Δ strains were transformed with the mtGFP plasmid and stained as described. During the exponential growth phase, we monitored the intracellular distribution of mitochondria and mtDNA from mother to daughter cells. In wild-type cells, actin filaments (red) displayed a polarized organization toward the budding daughter cell, with associated mitochondrial structures (green) and mtDNA nucleoids (blue) localized along this axis. In contrast, the *gcn5*Δ mutant exhibited disrupted actin polarization with a consequent collapse of the mitochondrial network, resulting in defective mitochondrial distribution and mtDNA inheritance. Notably, *rho*° cells (lacking mtDNA) showed normal actin polarization and successful mitochondria migration into the bud, despite the absence of DNA within the organelle.

#### 2.1.2. The *gcn5*Δ Strain Undergoes the Mitophagy Process

In several yeast mutants, mitochondrial dysfunction and morphological abnormalities can trigger the selective degradation of mitochondria via mitophagy. To investigate whether the *gcn5*Δ strain activates this pathway, we assessed mitophagy in the *gcn5*Δ under exponential growth conditions. As a positive control, wild-type cells were treated with Rapamycin, which is a known inducer of the mitophagy pathway in the exponential phase even in the presence of a fermentable carbon source [34].

Cells were transformed with a plasmid encoding GFP-Atg32, in which the mitophagy marker *ATG32* is fused to *GFP*. Upon mitophagy induction, Atg32 localized to the mitochondrial outer membrane, and the subsequent degradation of mitochondria results in the accumulation of free GFP within the vacuole. Vacuoles were labelled with the lipophilic dye FM4-64 (red), which allowed visualization of the GFP signal (green) co-localizing with vacuolar structures.

**Figure 2:**
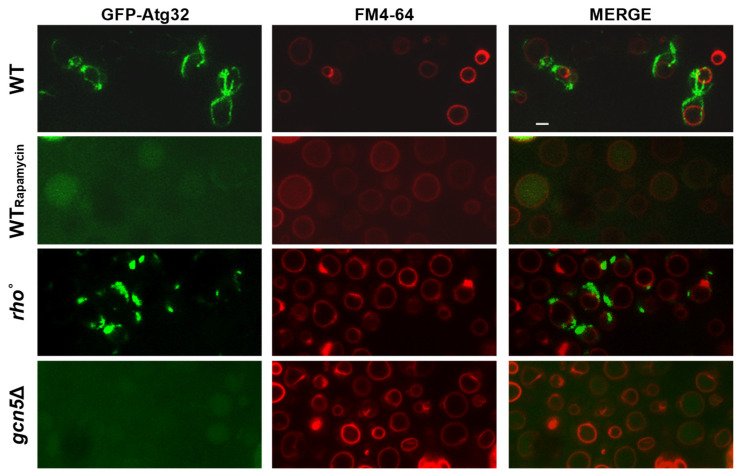
In *gcn5*Δ, the mitochondrial defects induce the mitophagy detected by fluorescence microscopy. Wild-type (WT), Rapamycin-treated WT, and *gcn5*Δ and *rho*° strains have been transformed with GFP-Atg32, which highlights mitochondrial membranes (green), and stained with FM4-64, which stains vacuolar membranes (red). Mitophagy is visible where the green GFP signal is localized within the vacuoles. Scale bars: 2 μm.

As shown in Figure 2, the *gcn5*Δ mutant cells displayed clear co-localization of the green and red signals, indicating active mitophagy in the cells. This pattern closely resembled that of the Rapamycin-treated wild-type cells control. In contrast, the *rho*° strain, which lacks mtDNA, showed no detectable accumulation of GFP in the vacuole, suggesting an absence of mitophagic activity under the same conditions.

These results suggest that the mitochondrial defects in the *gcn5*Δ strain are sufficient to activate the mitophagy pathway, highlighting a role for Gcn5 in maintaining mitochondrial integrity and preventing organelle turnover.

### 2.2. CSN5 Deletion Rescues the Mitochondrial Defects of gcn5Δ Strain

#### 2.2.1. Mitochondrial Phenotype of *csn5*Δ/*gcn5*Δ Double Mutant

**Figure 3:**
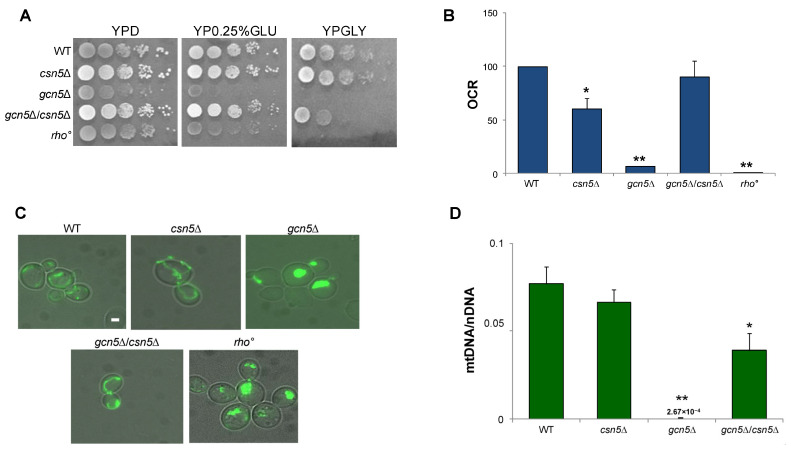
The deletion of *CSN5* gene suppresses the mitochondrial phenotypes of *gcn5*Δ. (**A**) Growth of serial dilutions of wild-type (WT) and *csn5*Δ, *gcn5*Δ, *gcn5*Δ/*csn5*Δ, and *rho*° strains in solid medium containing 2% glucose (fermentation), 0.25% glucose (intermediate), or 3% glycerol (respiration) as a carbon source. Images were taken after three days of growth at 28 °C. (**B**) Oxygen consumption rate (OCR) of strains grown overnight in 2% glucose-containing medium. (**C**) Mitochondrial network of strains transformed with mtGFP was observed by fluorescence microscopy (green). Scale bars: 1 μm. (**D**) qRT-PCR analysis of mtDNA level of the WT and derivative deleted mutants grown in 2% glucose-containing medium. The ratio between nuclear DNA (nDNA) mean value and mtDNA mean value (*OXI1*/*ACT1*) was used to overcome the variability among samples caused by total DNA quality. Data derive from at least three independent experiments and statistical significance by Student’s *t*-test is indicated. ** *p* < 0.01; * *p* < 0.05 for mutants versus WT strain.

To test the hypothesis that the CSN complex plays a role in suppressing the mitochondrial defects observed in the *gcn5*Δ strain, we constructed a double mutant by crossing isogenic strains deleted for *GCN5* and *CSN5* (see Section 4). As reported in Figure 3A, growth of the *gcn5*Δ mutant on a glycerol-containing medium, a non-fermentable carbon source requiring mitochondrial respiration, was partially rescued by the deletion of *CSN5*. Furthermore, growth on low glucose (0.25%), which favours respiratory metabolism by alleviating glucose repression, was fully restored in the double mutant, indicating a suppression of the respiratory growth defect. Oxygen consumption measurements (Figure 3B and Figure S1) revealed that the respiratory activity of the *csn5*Δ/*gcn5*Δ double mutant was comparable to that of the wild-type strain, supporting this observation. These findings demonstrate that the deletion of *CSN5* restores mitochondrial respiratory function in the *gcn5*Δ background. Mitochondrial morphology was also examined by fluorescence microscopy (Figure 3C). While the *gcn5*Δ mutant displayed a collapsed and fragmented mitochondrial network, reminiscent of the morphology observed in *rho*° cells, the *csn5*Δ/*gcn5*Δ double mutant exhibited a restored, wild-type-like mitochondrial structure.

Finally, qRT-PCR analysis of the mtDNA copy number (Figure 3D) showed a significant increase in the *csn5*Δ/*gcn5*Δ double mutant compared to the *gcn5*Δ mutant alone. This indicates partial recovery of mitochondrial genome stability.

Taken together, these results demonstrate that the deletion of *CSN5* compensates for multiple mitochondrial defects associated with the loss of the *GCN5* gene, including impaired respiration, defective morphology, and mtDNA instability.

#### 2.2.2. *CSN5* Deletion Enhances Mitochondrial Stability and Suppresses Mitophagy in the *gcn5*Δ Background

**Figure 4:**
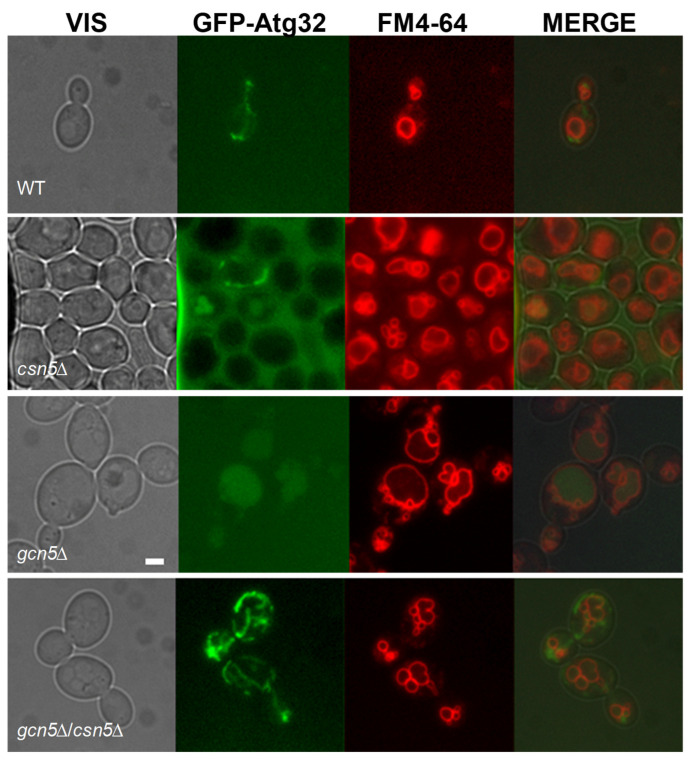
The deletion of *CSN5* suppresses the mitophagy in *gcn5*Δ. Fluorescence microscopy of wild-type (WT) and *csn5*Δ, *gcn5*Δ, and *gcn5*Δ/*csn5*Δ strains transformed with GFP-Atg32, which highlights mitochondrial membranes (green), and stained with FM4-64 (vacuolar membranes in red). Scale bars: 2 μm.

As shown in Figure 2, the severe mitochondrial dysfunction in the *gcn5*Δ strain activates the mitophagy pathway. To determine whether deletion of *CSN5* can suppress this response, we analyzed mitophagy in the *csn5*Δ/*gcn5*Δ double mutant using the GFP-Atg32 reporter system. As shown in Figure 4, the double mutant exhibited no evidence of mitophagy activation under the conditions tested, in contrast to the pronounced mitophagy observed in *gcn5*Δ cells. The absence of the GFP signal co-localization with the vacuolar marker FM4-64 indicates that mitochondrial turnover is no longer triggered in the double mutant. These findings demonstrate that loss of the *CSN5* function, which affects the ubiquitin–proteasome system, is sufficient to suppress the morphological and functional mitochondrial defects, as well as the downstream activation of mitophagy in the *gcn5*Δ mutant. This suggests that *CSN5* plays a regulatory role in mitochondrial quality control and stability, particularly under conditions of Gcn5 deficiency.

### 2.3. Ergosterol Supplementation Restores Mitochondrial Functionality

#### 2.3.1. Ergosterol Content Is Decreased in Mutant Cells

**Figure 5:**
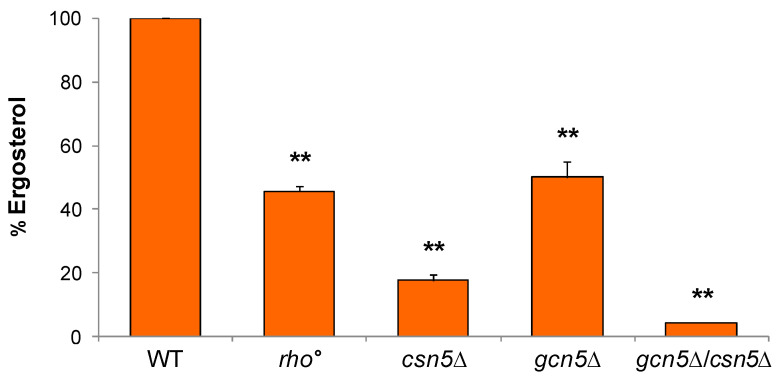
Ergosterol content in mutant strains. The esterified ergosterol was measured by Hig- Performance Liquid Chromatography (HPLC) analysis in wild-type (WT) and *rho*°, *gcn5*Δ, *csn5*Δ, and *gcn5*Δ/*csn5*Δ cells. Values were reported as ergosterol percentage in the mutants compared to that of wild-type cells. Data derive from at least three independent experiments and statistical significance by Student’s *t*-test is indicated. ** *p* < 0.01; for mutants versus WT strain.

Previous studies have demonstrated that ergosterol levels are significantly reduced in *csn5*Δ compared with the wild-type strain, and that ergosterol is essential for anchoring mtDNA in the lipid rafts microdomains within the inner mitochondrial membrane [22]. To evaluate whether alterations in ergosterol biosynthesis contribute to the mitochondrial phenotypes observed in our mutants, we quantified the ergosterol content of wild-type and mutant strains using High-Performance Liquid Chromatography (HPLC). As shown in Figure 5, all mutant strains displayed a significant reduction in ergosterol content relative to the wild-type. Consistent with prior findings [22], ergosterol levels were reduced to approximately 20% in *csn5*Δ cells and to around 50% in *rho*° cells. Notably, the *gcn5*Δ mutant also exhibited a ~50% reduction in ergosterol levels. Interestingly, the *csn5*Δ/*gcn5*Δ mutant showed an approximately 95% decrease in ergosterol content compared to the wild-type, indicating a possible additive or synergistic effect of the combined gene deletions on ergosterol biosynthesis.

#### 2.3.2. Ergosterol Rescues Mitochondrial Defects of gcn5Δ Mutant and Acts Additively with CSN5 Deletion

**Figure 6:**
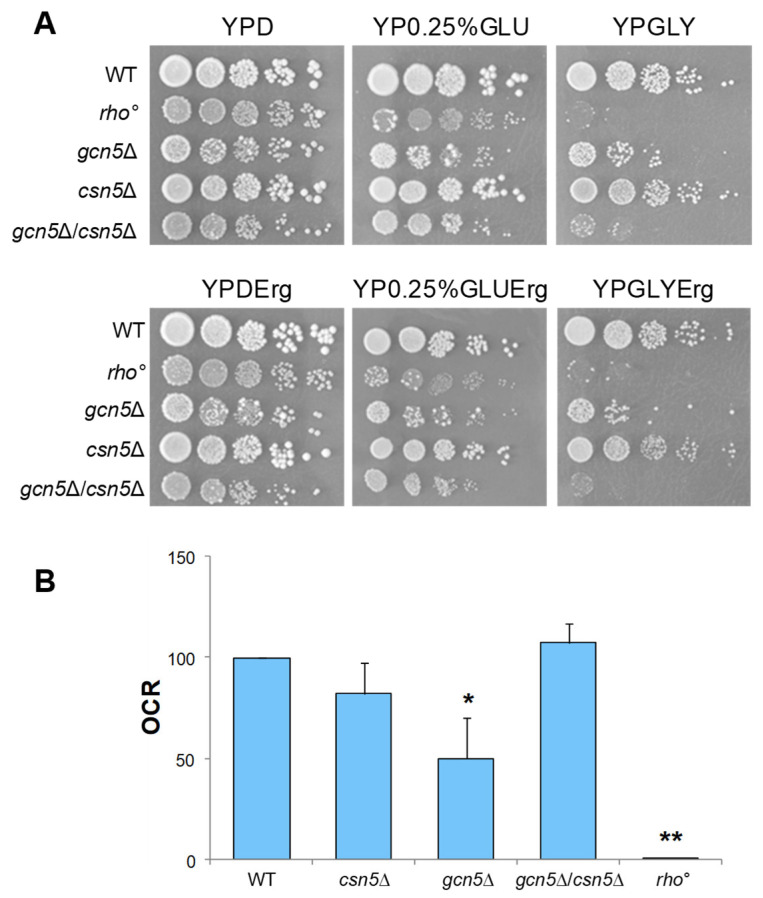
Respiration phenotypes of the mutant strains treated with ergosterol. (**A**) Growth of wild-type (WT), *rho*°, *gcn5*Δ, *csn5*Δ, and *gcn5*Δ/*csn5*Δ mutants in a solid medium (serial dilutions) containing 2% glucose, 0.25% glucose, or 3% glycerol as a carbon source (upper panels) and on the same media supplemented with 0.02 mg/mL ergosterol. All strains were grown overnight in 2% glucose-liquid-containing medium with the addition of 0.02 mg/mL ergosterol before being plated on solid media. Images were taken after three days of growth at 28 °C. (**B**) Oxygen consumption rate (OCR) of indicated strains grown in 2% glucose-containing medium with the addition of 0.02 mg/mL ergosterol. Data derive from at least three independent experiments and statistical significance by Student’s *t*-test is indicated. ** *p* < 0.01; * *p* < 0.05 for mutants versus WT strain.

The markedly reduced ergosterol content observed in the *gcn5*Δ*/csn5*Δ double mutant prompted us to further explore the contribution of ergosterol to mitochondrial function in these strains. To this end, we assessed the respiratory phenotypes of the mutant strains following ergosterol supplementation. Cells were grown in a glucose-containing liquid medium supplemented with 0.02 mg/mL ergosterol, as described in the Section 4 [11]. Figure 6A (upper panels) shows the growth of the mutants in a solid medium under fermentative, respiratory, or intermediate condition. In the bottom panels, the same cells were grown on the same solid medium as in the upper panels, but with additional ergosterol supplementation. In both cases, we observed that the ergosterol treatment increased the growth capability of the *gcn5*Δ mutant in both low glucose and glycerol respiratory media. Growth recovery was evident when compared with the growth of the untreated *gcn5*Δ cells (Figure 2), indicating a positive effect of ergosterol on respiratory growth. Ergosterol supplementation also improved the respiration capability of all deleted mutants (Figure 6B and Figure S2A).

**Figure 7:**
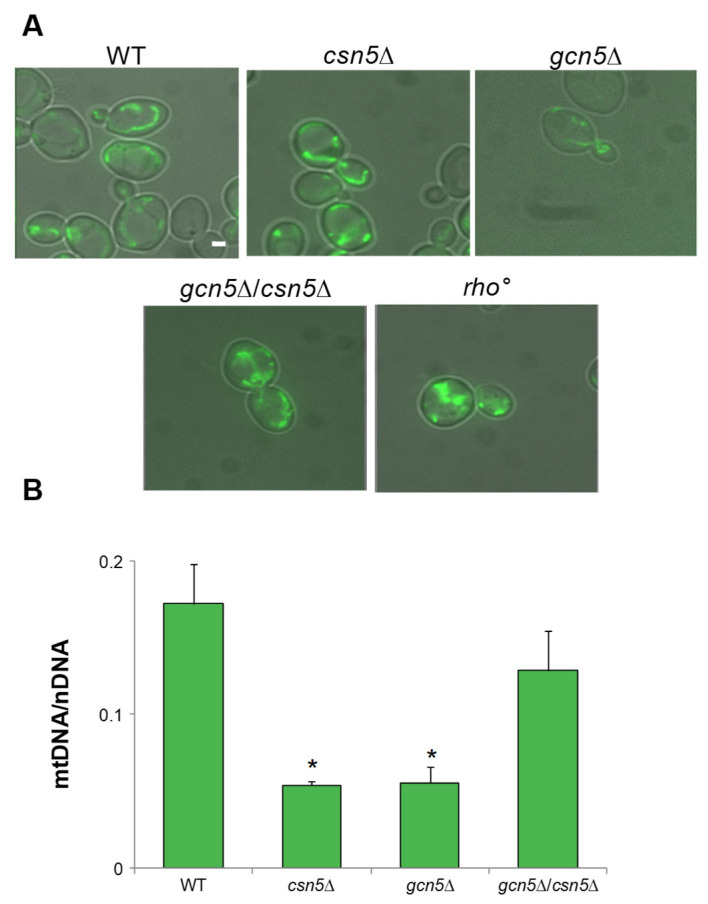
Ergosterol supplementation suppresses the mitochondrial morphology defects and the mtDNA instability. (**A**) Fluorescence microscopy of wild-type (WT) and *rho*°, *gcn5*Δ, *csn5*Δ, and *gcn5*Δ/*csn5*Δ mutants grown in 2% glucose medium with the addition of 0.02 mg/mL ergosterol. The strains were transformed with mtGFP to visualize the mitochondrial network (green). Scale bars: 1 μm. (**B**) qRT-PCR analysis of mtDNA level of the wild-type and the mutants grown as in (**A**). The ratio between nuclear DNA (nDNA) mean value and mtDNA mean value (*OXI1*/*ACT1*) was used to overcome the variability among samples caused by total DNA quality. Data derive from at least three independent experiments and statistical significance by Student’s *t*-test is indicated. * *p* < 0.05 for deleted versus WT strain.

Ergosterol also improved the mitochondrial morphology of the *gcn*5Δ strain, resulting in a tubular mitochondrial network similar to that of the wild-type strain (Figure 7A). Additionally, stabilization of the mtDNA content was also observed in the mutants after treatment (Figure 7B and Figure S2B).

These results demonstrate that ergosterol exerts a suppressive effect on *gcn5*Δ mitochondrial defects that is similar to that of *CSN5* gene deletion. Furthermore, the improvement in mitochondrial function observed in all mutants following ergosterol treatment further supports the hypothesis that ergosterol may have an additive effect with the *CSN5* deletion.

## 3. Discussion

In this study we initially characterized the mitochondrial phenotypes associated with the deletion of the *GCN5* gene in the yeast *Saccharomyces cerevisiae*, with the aim of identifying the underlying mechanism of mtDNA instability. We observed significant defects in mitochondrial inheritance due to the depolarization of the actin filaments in the *gcn5*Δ mutant. This prevented the proper migration of mitochondria, and consequently of the mtDNA, from the mother cell to the daughter cell during mitosis. Actin filament polarization is crucial for mitochondrial inheritance in yeast [35]; notably, *rho*° cells, which lack mtDNA, exhibit normal actin polarization and mitochondrial distribution, emphasizing that actin depolarization is specifically linked to *GCN5* deletion [36]. Furthermore, *gcn5*Δ cells exhibit disrupted mitochondrial morphology and progressive mtDNA loss, which correlates with increasingly severe respiratory dysfunction over successive cell divisions [5,6]. These overall mitochondrial defects collectively trigger the activation of the mitophagy pathway, which involves the selective removal of impaired mitochondria via vacuolar degradation in yeast (lysosomal in mammals) [37]. Thus, our findings underscore the critical role of the *GCN5* gene in maintaining mitochondrial integrity and function in yeast.

In order to identify pathways that can suppress mitochondrial defects in the *gcn5*Δ strain, we examined the genetic interactions between *GCN5* and the components of the ubiquitin–proteasome pathway, with a particular focus on the COP9 signalosome (CSN) complex. The ubiquitin–proteasome system is intricately connected with epigenetic regulation, protein stability, and the modulation of cellular responses. However, dissecting its specific mitochondrial roles remains challenging due to pathway complexity [2]. We targeted *CSN5*, encoding a non-essential subunit of the CSN complex which does not exhibit mitochondrial dysfunction upon deletion, unlike many other proteasomal mutants [15,38]. Earlier studies have suggested possible genetic and functional interactions between the SAGA complex and the CSN, particularly involving *GCN5*. One proposed link arose from observations involving Csn12, a protein originally annotated as a putative CSN subunit in yeast [20]. However, more recent biochemical analyses have demonstrated that Csn12 does not stably associate with the canonical CSN complex [39]. Instead, Csn12 has been implicated in mRNA splicing and RNA processing, which may explain its genetic interactions with transcription-related factors such as Gcn5. These findings highlight the complexity of regulatory networks governing gene expression and the importance of revisiting earlier subunit assignments based on updated functional and structural data.

Overall, the results demonstrated that the *csn5*Δ/*gcn5*Δ double mutant no longer exhibited the mitochondrial defects observed in the *gcn5*Δ single mutant. Therefore, the *GCN5* and *CSN5* genes are both involved in the regulation of mitochondrial functionality, albeit in different ways. *GCN5*, coding for an acetyltransferase, can influence the expression of genes involved in mitochondrial processes through chromatin modification, favouring the activation of genes necessary for mitochondrial physiology. Gcn5 is required to activate nuclear genes that compensate for impaired mitochondrial function. This process, known as retrograde regulation, is critical for communication between mitochondria and the nucleus in order to regulate gene expression in the case of mitochondrial dysfunctions. Indeed, Gcn5 is also a part of the SAGA/SLIK transcriptional activator complex, which includes the retrograde factor Rtg2. This factor is involved in regulating the expression of genes that code for mitochondrial, cytoplasmic, and peroxisomal proteins, as well as cellular responses to mitochondrial damage [40]. In human cells, the post-translational modifications of mitochondrial proteins due to epigenetic factors of the SAGA complex, represent an interesting regulatory mechanism that links mitochondria and re-programmed metabolism in cancer cells [41]. Furthermore, the mitochondrial homologue of the nuclear *GCN5* (*GCN5L1*) has been demonstrated to regulate mitochondrial proteins through acetylation. It is also involved in mitochondrial metabolism, biogenesis, and mitophagy [42].

The role of ergosterol in mitochondrial function was also investigated in this work. Previous studies have demonstrated the involvement of Csn5 in the ergosterol biosynthetic pathway [21], as well as the key role of the ergosterol content in the cells in mtDNA stability and maintenance [11]. There is an interplay between mitochondrial function and the ergosterol biosynthetic pathway. In *S. cerevisiae*, ergosterol is an essential component of all cell membranes, including mitochondrial membranes, and it is found at its highest concentration in the inner mitochondrial membrane [43]. The adhesion of mtDNA, inside the nucleoids, to this membrane is related to lipid raft-like mitochondrial microdomains, that are enriched in ergosterol, sphingolipids, and proteins, which are constantly exchanged via the endoplasmic reticulum–mitochondria complex [44,45]. This ensures the correct mitochondrial morphology and the maintenance of mtDNA [11]. It is therefore possible that the low ergosterol content found in the *gcn5*Δ mutant contributes to the instability of its mtDNA. The SAGA complex has also been shown to play a role in ergosterol biosynthesis [46]. Indeed, it has been reported that the SAGA complex acts as a coactivator of ergosterol transcription, inducing specific changes to the transcriptional profile, in response to variations in cellular sterol composition. This could account for the low ergosterol levels observed in the *gcn5*Δ mutant [47]. The addition of ergosterol to the growth medium improved the mitochondrial functionality of the *gcn5*Δ strain. The improvement in respiratory growth and oxygen consumption rate were associated with greater mtDNA stability and the restoration of a normal mitochondrial network. These data are consistent with a strong link existing between ergosterol and acetyl-CoA and, thus, with Gcn5 activity. Indeed, the first step of ergosterol biosynthesis involves the formation of mevalonate from acetyl-CoA itself [48]. Furthermore, the cellular acetyl-CoA availability efficiently regulates the Gcn5 lysine-acetyltransferase activity [49,50]. In the *csn5*Δ/*gcn5*Δ double mutant, the ergosterol content was significantly lower than in the wild-type strain. We hypothesize that this low amount may be due to the combined effect of deleting the *CSN5* and *GCN5* genes and the synergistic involvement of these two genes in regulating ergosterol biosynthesis. The *CSN5* deletion rescued the defective phenotype caused by the *GCN5* deletion, improving the mitochondrial defects despite the low level of ergosterol in the double mutant. Adding ergosterol then worked in synergy with the double deletion, improving the mitochondrial phenotypes analyzed here.

In conclusion, our results demonstrated a genetic interaction between *GCN5* and *CSN5* in the yeast *S. cerevisiae*. This interaction links the mechanisms of epigenetic regulation and protein stability, which are mediated by the SAGA and COP9 signalosome complexes, respectively, in order to ensure mitochondrial integrity. Recovery of mitochondrial function in the absence of the two genes is also favoured by ergosterol supplementation, suggesting that Csn5-mediated restoration is associated with its regulatory function in the ergosterol biosynthetic pathway. Further studies will be needed to elucidate the roles and the mechanisms of the interaction between *GCN5* and *CSN5*, as well as the potential involvement of additional factors within their respective complexes. Future research will also focus on studying the link between protein acetylation and degradation. It has been reported that proteins with free α-amino groups can undergo ATP-dependent ubiquitin degradation, a process that can be prevented by acetylating the N-terminal α-amino group [51]. It would be interesting to determine whether non-acetylated proteins could undergo ubiquitylation for degradation in the absence of Gcn5. Furthermore, it should be clarified whether the absence of Csn5 in the double mutant could prevent the activation of E3 ligases, resulting in the accumulation of undegraded proteins inside the cell. Therefore, further research will be needed to establish whether Gcn5 and Csn5-regulated E3 ligases act on the same substrates.

## 4. Materials and Methods

### 4.1. Yeast Strains and Growth Conditions

*Saccharomyces cerevisiae* strains used in this work are derived from a W303-1A nuclear background. The complete list is reported in Table 1. The *rho*° strain (completely lacking mtDNA) was obtained from isogenic rho^+^ strain (having a wild-type mtDNA) by Ethidium Bromide treatment [52].

The *csn5*Δ/*gcn5*Δ mutant was generated by mating, sporulation, and tetrad dissection. The single *csn5*Δ and *gcn5*Δ strains were crossed and, after sporulation of the diploid strain, tetrad dissection was performed. The double deleted mutant was obtained by selection on the G148 plate of the tetrad in which two spores were kanamycin sensitive and two spores showed a kanamycin-resistant recombinant phenotype.

Yeast cells were grown at 28 °C in YP medium (1% yeast extract, 2% bactopeptone) containing 2% glucose (YPD) or 0.25% glucose, either with or without 0.02 mg/mL ergosterol supplementation (Sigma-Aldrich, St. Louis, MO, USA), or 3% glycerol. For the selection of the double mutant, a YPD medium was supplemented with 0.1 mg/mL G418 (Sigma-Aldrich, St. Louis, MO, USA). Agarose was added in the solid medium at 2% concentration.

Comparison of growth capability was investigated by serial dilutions of concentrated suspensions (5–9 × 10^6^ cell/mL), prepared from fresh single colonies after overnight incubation at 28 °C and spotted onto a plate. Pictures were acquired after 3 days of growth at 28 °C.

### 4.2. Oxygen Consumption Measurement

Respiration studies were performed using Clark Oxygen Electrode (Hansatech Instruments, King’s Lynn, UK) [55]. A total of 0.03 g of yeast cells (wet weight) were collected after growth in the YPD medium (with or without 0.02 mg/mL ergosterol), washed with 1 mL sterile water, and resuspended in 1 mL of 10 mM Na-phosphate buffer pH 7.4 containing 20 mM glucose. Samples were loaded in the Reaction Vessel of a previously calibrated Oxygen Electrode Chamber.

### 4.3. Plasmids and Transformation Experiments

DNA manipulation, restriction enzyme digestion, plasmid preparations, as well as *Escherichia coli* and yeast transformations, were performed as described in [56].

Plasmids used to transform the yeast strains were: (i) mtGFP, the episomal plasmid pVT100U in which the gene coding for the Green Fluorescent Protein (fused at the 3′end with the mitochondrial targeting sequence of subunit 9 of the *Neurospora crassa* ATP-ase) is cloned [57]; (ii) GFP-Atg32, the centromeric plasmid pRS416 in which the gene *ATG32* is fused at 5′end with the *GFP* gene [58].

### 4.4. Quantification of mtDNA

Quantitative Real Time-PCR (qRT-PCR) was performed as previously described to quantify the mtDNA copy number [5]. For DNA extraction yeast cells were collected after overnight growth in the YPD medium. The same samples were also grown in the YPD medium with 0.02 mg/mL ergosterol. The sequences of the oligonucleotides OXI1 For and OXI1 Rev for mitochondrial gene amplification or ACT1 For and ACT1 Rev for nuclear gene amplification are reported in Table 2.

### 4.5. Actin Detection

To visualize the cellular actin filaments into the cells, 100 μL of transformed cells with the mtGFP plasmid were collected at the exponential phase after overnight growth in the YPD medium. The cells were fixed for 45 min at room temperature by adding two volumes of 1% formaldehyde. The samples were centrifuged for 5 min at 3500 rpm and the cells were resuspended in 50 μL of Triton X-100 (0.1%). After 10 min at room temperature, 6.6 μM Rhodamine-Phalloidin dye (Invitrogen, Thermo Fisher Scientific, Waltham, MA, USA) was added. After 45 min of incubation at room temperature, the cells were washed with sterile water and resuspended in 5 μL of DAPI staining (1 μg/mL, Sigma-Aldrich, St. Louis, MO, USA). The observation was carried out by fluorescence microscope.

### 4.6. Mitophagy Detection

To selectively detect the mitophagy, 1 mL of transformed cells with the GFP-Atg32 plasmid were collected at the exponential phase after overnight growth in the YPD liquid medium, and the FM4-64 vacuolar lipophilic dye (Invitrogen, Thermo Fisher Scientific, Waltham, MA, USA) was added to 16 μM final concentration. After 1 h of incubation at 28 °C, the cells were washed with 1 mL YPD medium twice. The cells were resuspended in 2 mL YPD and incubated for 2 h at 28 °C before observation by fluorescence microscope [59]. To obtain a positive control, a sample of wild-type cells was treated with Rapamycin, an inducer of mitophagy: the cells are resuspended in 2 mL YPD + 30 μL of Rapamycin (3 μg/mL, Sigma-Aldrich, St. Louis, MO, USA) before incubation for 2 h at 28 °C.

### 4.7. Ergosterol Extraction

Evaluation of the ergosterol amount in yeast cells was performed as previously described with minor changes [60]. For each yeast strain, 20 OD_600_ of an overnight-grown culture in a glucose-containing medium was harvested. The cell suspension was then collected into a 2 mL screw cap tube and washed once with sterile distilled water. Finally, the cell pellet was suspended in 600 μL of an ethanol/KOH solution. The hydroxide solution was prepared by mixing 2.5 g KOH with 3.6 mL DW and adding ethanol up to 10 mL. An amount of 50 mL of a solution of ethanolic butylated hydroxytoluene (BHT) (10 mg BHT in 1 mL ethanol) was added to prevent ergosterol oxidation. The suspension was incubated at 80 °C in a heat block under constant shaking for 1 h. Next, 600 μL of *n*-heptane and 55 μL of distilled water were added serially to the mix, followed by vigorous vortexing for 3 min. The samples were left for 5 min at room temperature for phase separation; then the upper phase was transferred to a new Eppendorf and dried under a nitrogen flow; and the samples were resuspended in methanol (MeOH) for analysis in HPLC.

### 4.8. High-Performance Liquid Chromatography (HPLC) Analysis

The quantification of ergosterol extracted from yeast cells as described in the previous paragraph was performed by a HPLC-UV/diode array detector (DAD). The analyses were performed following the method described by [61] with minor modifications. The liquid chromatography (Agilent 1260 Infinity, Agilent Technologies, Santa Clara, CA, USA) was equipped with a quaternary gradient pump, an autosampler injection system, a column oven set at 25 °C, a DAD, and a chromatography data system (ChemStation version C.01.07). A Poroshell-120 C18 column (4.6 × 50 mm, 2.7 μm particle sizes, Agilent Technologies, Santa Clara, CA, USA) was used. The mobile phase was an isocratic mixture of MeOH and acetonitrile (CH_3_CN) (20:80 *v/v*) eluting at a flow rate of 1.0 mL/min for 10 min. Ergosterol was identified in cell extracts by comparing the retention time and the UV spectra of the peak areas recorded in the chromatogram with those of the authentic standard. UV spectra were recorded in the range of 200 to 450 nm. Quantification of ergosterol was performed according to the external standard method, integrating peak areas acquired at the wavelength corresponding to its maximum absorbance (280 nm), at the retention time of the corresponding ergosterol standard. Stock solution of ergosterol was prepared in amber glass at a concentration of 50 ng/mL, and work solutions were prepared by appropriately diluting the stock solution with MeOH. After use, the solutions were stored at −25 °C.

### 4.9. Fluorescence Imaging

Cells were visualized by fluorescence microscopy on Zeiss Axio Imager Z1 Fluorescence Microscope (Carl Zeiss, Oberkochen, Germany). Images were acquired using Axio-Vision 4.8 Digital Image Processing System (Carl Zeiss, Oberkochen, Germany) and objective lens 63× oil.

### 4.10. Statistical Analysis

Experiments were performed at least in triplicate. Data are presented as mean ± SE and Student’s *t* test were used to determine the statistical significance between experimental groups. Statistical significance was defined as * *p* < 0.05, ** *p* < 0.01.

## Figures and Tables

**Table 1:** *Saccharomyces cerevisiae* strains used in this work.

Strain	Genotype	Reference
W303-1A rho^+^	MATa ade2-1 trp1-1 leu2-3,112 his3-11,15 ura3-1 can1-100 ssd1 rho^+^	[53]
W303-1A *rho*°	MATa ade2-1 trp1-1 leu2-3,112 his3-11,15 ura3-1 can1-100 ssd1 *rho*°	[5]
*gcn5*Δ rho^+^	MATa ade2-1 trp1-1 leu2-3,112 his3-11,15 ura3-1 can1-100 ssd1 gcn5::KanMX4 rho^+^	[54]
*csn5*Δ rho^+^	MATa ade2-1 trp1-1 leu2-3,112 his3-11,15 ura3-1 can1-100 ssd1 csn5::KanMX4 rho^+^	[21]
*csn5*Δ rho^+^	MATα ade2-1 trp1-1 leu2-3,112 his3-11,15 ura3-1 can1-100 ssd1 csn5::KanMX4 rho^+^	This work
*csn5*Δ*/gcn5*Δ rho^+^	ade2-1 trp1-1 leu2-3,112 his3-11,15 ura3-1 can1-100 ssd1 *csn5*Δ*/gcn5*Δ rho^+^	This work

**Table 2:** Oligonucleotides used in this work.

Oligonucleotides	Sequences
OXI1 For	GTACCAACACCTTATGCAT
OXI1 Rev	CATTCAAGATACTAAACCTAA
ACT1 For	ACGTTCCAGCCTTCTACGTTTCCA
ACT1 Rev	AGTCAGTCAAATCTCTACCGGCCA

## Data Availability

The original contributions presented in this study are included in the article and Supplementary Materials. Further inquiries can be directed to the corresponding author.

## Supplementary Materials

The following supporting information can be downloaded at: https://www.mdpi.com/article/10.3390/ijms26146916/s1.

## Abbreviations

The following abbreviations are used in this manuscript:

SAGA	Spt-Ada-Gcn5 Acetyltransferase
KAT	Lysine-Acetyltransferase
HAT	Histone Acetyltransferase
DUB	Deubiquitinase
SLIK	SAGA-like
OMM	Outer Mitochondrial Membrane
IMM	Inner Mitochondrial Membrane
mtDNA	Mitochondrial DNA
nDNA	Nuclear DNA
CSN	COP9 Signalosome
CRLs	Cullin RING E3 Ligases
UFA	Unsaturated Fatty Acids
ER	Endoplasmic Reticulum
YPD	Yeast Extract + Peptone + Glucose
mtGFP	Mitochondrial Green Fluorescent Protein
qRT-PCR	Quantitative Real Time Polymerase Chain Reaction
DAPI	4′,6-diamidino-2-phenylindole
FM4-64	*N*-(3-Triethylammoniumpropyl)-4-(6-(4-(Diethylamino) Phenyl) Hexatrienyl) Pyridinium Dibromide
OD_600_	Optical Density at 600 nm
HPLC	High Performance Liquid Chromatography
DAD	Diode Array Detector
BHT	Butylated Hydroxytoluene
SE	Standard Error
OCR	Oxygen Consumption Rate
