# Supplementary material for: Csn5 Depletion Reverses Mitochondrial Defects in GCN5-Null Saccharomyces cerevisiae
Source: Int J Mol Sci. 2025 Jul 18;26(14):6916. doi: 10.3390/ijms26146916 (PMC12294998; doi:10.3390/ijms26146916)
Supplement: Supplementary file 1 [file ijms-26-06916-s001.zip › ijms-3760799-supplementary.pdf]

Supplementary Materials

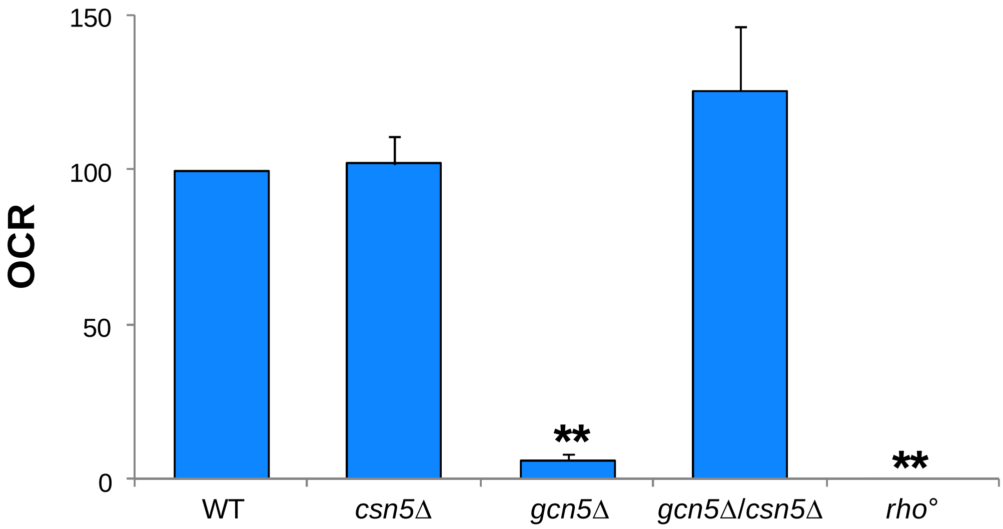

Figure S1. Respiration capability of yeast strains grown in low glucose. Oxygen consumption rate (OCR, expressed as O_2 nmol/mL) of wild-type (WT), *csn5*Δ, *gcn5*Δ, *gcn5*Δ/*csn5*Δ and *rho*° mutants grown overnight in 0.25% glucose containing medium. Data derive from at least three independent experiments and statistical significance by Student's t-test is indicated. ** $p<0.01$; for mutants versus WT strain.

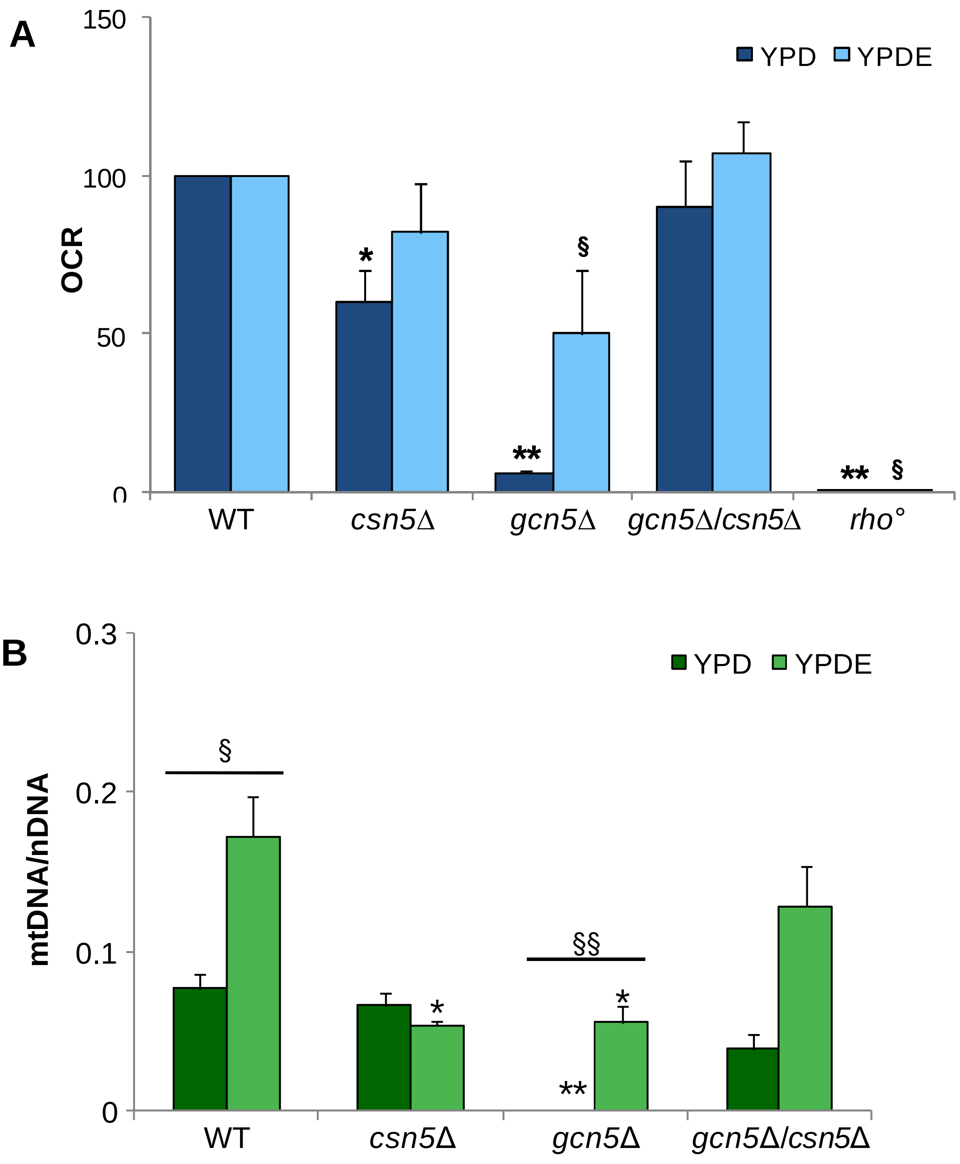

Fig. S2 (A) Comparison of respiration capability and mtDNA stability of yeast strains grown in YPD and YPDE media. Oxygen consumption rate (OCR, expressed as O_2 nmol/mL) of wild-type (WT), *csn5*Δ, *gcn5*Δ, *gcn5*Δ/*csn5*Δ and *rho*° mutants grown in the presence (YPDE) or absence (YPD) of ergosterol (0.02 mg/mL). Data derive from at least three independent experiments and statistical significance by Student's t-test is indicated. ** $p<0.01$; * $p<0.05$ for mutants versus WT strain. § $p<0.05$ for YPDE versus YPD grown cells. **(B)** mtDNA copy number of the WT and derivative deleted mutants grown in same media as before, and obtained by qRT-PCR analysis. The ratio between nuclear DNA (nDNA) mean value and mtDNA mean value (*OXI1*/*ACT1*) was used to overcome the variability among samples caused by total DNA quality. Data derive from at least three independent experiments and statistical significance by Student's t-test is indicated. ** $p<0.01$; * $p<0.05$ for deleted versus WT strain. §§ $p<0.01$; § $p<0.05$ for YPDE versus YPD grown cells.
